# When crust comes of age: on the chemical evolution of Archaean, felsic continental crust by crustal drip tectonics

**DOI:** 10.1098/rsta.2018.0103

**Published:** 2018-10-01

**Authors:** O. Nebel, F. A. Capitanio, J.-F. Moyen, R. F. Weinberg, F. Clos, Y. J. Nebel-Jacobsen, P. A. Cawood

**Affiliations:** 1 School of Earth, Atmosphere and Environment, Monash University, Clayton, 3800 Victoria, Australia; 2 Laboratoire Magmas et Volcans, Université de Lyon, UJM-UCA-CNRS-IRD, 23 rue Dr. Paul Michelon, 42023 Saint Etienne, France

**Keywords:** crustal chemistry, Archaean-proterozoic transition, plate tectonics, tonalite–trondhjemite–granodiorite

## Abstract

The secular evolution of the Earth's crust is marked by a profound change in average crustal chemistry between 3.2 and 2.5 Ga. A key marker for this change is the transition from Archaean sodic granitoid intrusions of the tonalite–trondhjemite–granodiorite (TTG) series to potassic (K) granitic suites, akin (but not identical) to I-type granites that today are associated with subduction zones. It remains poorly constrained as to how and why this change was initiated and if it holds clues about the geodynamic transition from a pre-plate tectonic mode, often referred to as stagnant lid, to mobile plate tectonics. Here, we combine a series of proposed mechanisms for Archaean crustal geodynamics in a single model to explain the observed change in granitoid chemistry. Numeric modelling indicates that upper mantle convection drives crustal flow and subsidence, leading to profound diversity in lithospheric thickness with thin versus thick proto-plates. When convecting asthenospheric mantle interacts with lower lithosphere, scattered crustal drips are created. Under increasing P-T conditions, partial melting of hydrated meta-basalt within these drips produces felsic melts that intrude the overlying crust to form TTG. Dome structures, in which these melts can be preserved, are a positive diapiric expression of these negative drips. Transitional TTG with elevated K mark a second evolutionary stage, and are blends of subsided and remelted older TTG forming K-rich melts and new TTG melts. Ascending TTG-derived melts from asymmetric drips interact with the asthenospheric mantle to form hot, high-Mg sanukitoid. These melts are small in volume, predominantly underplated, and their heat triggered melting of lower crustal successions to form higher-K granites. Importantly, this evolution operates as a disseminated process in space and time over hundreds of millions of years (greater than 200 Ma) in all cratons. This focused ageing of the crust implies that compiled geochemical data can only broadly reflect geodynamic changes on a global or even craton-wide scale. The observed change in crustal chemistry does mark the lead up to but not the initiation of modern-style subduction.

This article is part of a discussion meeting issue ‘Earth dynamics and the development of plate tectonics’.

## 1. Introduction

The young Earth's early lithosphere, comprising its outer crustal shell and underlying uppermost mantle, was forged during magma ocean solidification in the Hadean eon *ca* 4.4 Ga ago [1–3]. This first lithosphere likely formed as a stagnant lid separated by a thermal boundary layer from a vigorously convecting Hadean mantle [4]. Of this crust, only single zircon grains, predominantly from Australia's Narryer Gneiss Terrane [2,5,6] but also other cratons [7,8] remain, as well as perhaps the highly metamorphosed section in the Canadian Nuvvuagittuq greenstone belt [9]. Following this earliest stage of which little is known, Archaean passive stagnant-lid tectonics have been proposed to have shaped the Earth's surface, with a continuous crustal cover on top of a convecting mantle before this was replaced by active plate tectonics [10,11]. Plate tectonics with subduction zones at convergent plate margins has been the modus operandi of the planet throughout the Phanerozoic, Proterozoic and possibly part of the Late Archaean [12–15].

Independent of these models of tectonic regime, and based simply on observations from the rock record, a transition in Earth's history was first recognized in rocks of the Canadian shield and thought to have occurred at 2390 Ma [16]. It corresponds to the development of large sedimentary basins and abundant dike swarms and provides evidence for stabilization of a rigid lithosphere [17]. The overall character of this change, involving a change in crustal stability and their deformational and metamorphic histories, was recognized on other continents, defining the Archaean–Proterozoic boundary with an arbitrary age boundary of 2500 Ma [18].

Global chemical changes were soon recognized to occur at roughly the same time. Engel *et al*. [19] identified a change in K/Na in the crust around *ca* 2.5 Ga, Windley [20] later recognized that the ‘boundary’ is transitional and diachronous ranging from 3.0 to 2.5 Ga, and McLennan & Taylor proposed that most of the continental crust was forged by 2.5 Ga [21]. Condie [22] subsequently noted that this transition also corresponded to a change in basalt chemistry. With the wealth of geochemical data available today, this shift in the composition of the crust has been shown to extend globally [17]. Combined geochemical and chronological studies on Archaean terranes demonstrate a broadly defined but marked change in crustal chemistry in the Mid to Late Archaean eon between 3.2 and 2.5 Ga [23–27]. The application of statistical geochemistry to the wealth of data published over decades has, therefore, confirmed a change in the way crust is either produced or reworked. Notable is that this change in crustal rock chemistry towards more felsic, ‘continental’ crust is complemented by a development in shale geochemistry that sample larger, upper crustal areas through crustal reworking and weathering [28]. These shales clearly record reworking from 3 Ga onwards, as opposed to juvenile input into the crust prior to 3 Ga. A shift from mafic to felsic trace element composition is also recorded in ocean-floor sediments from 3.2 to 2.5 Ga [29,30]. Combining all of these records plausibly mirror a transition in the way crustal dynamics operated around that time.

**Figure 1.**
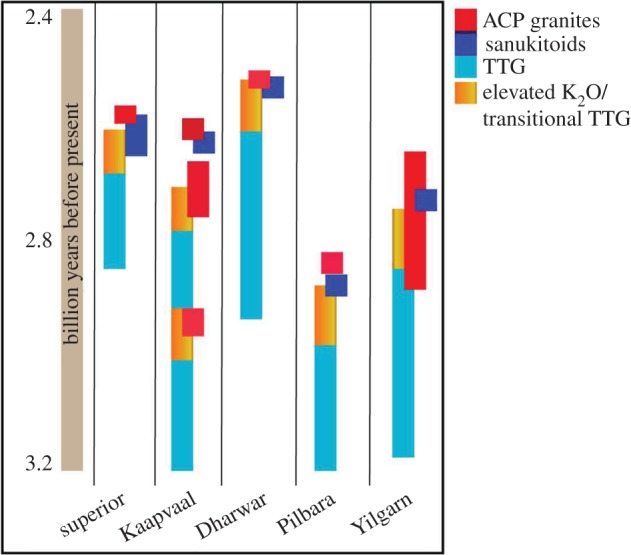
Schematic compilation of temporal distributions of various Archaean granitoids, classified according to their rock types as defined in this contribution to illustrate the temporal offset in changing chemistry in different cratons. Modified after Laurent *et al*. [37]. (Online version in colour.)

A geodynamic change from passive to active plate tectonics would have imposed profound changes in physical appearance of the Earth's crust (and topography), but also in the modes of melting of lower crust versus mantle wedge (in non- versus Archaean-style subduction) and with this the chemical composition of the crust [24,25]. The chemistry of the preserved rock record cannot directly be linked to geodynamics, but holds clues on the source and conditions of melting of this source, and thus allows inferences on the tectonic environment of formation of igneous rocks, although interpretations are often non-unique [31–36]. On the modern Earth, melting of the mantle above subduction zones, along with recycling and reworking of the crust at convergent plate margins, leave an indelible imprint on the continental crust. Whether the earliest felsic, continental crust was indeed formed in subduction zones, or not, can thus be tested using rock chemistry. A change in rock chemistry between 3.0 and 2.5 Ga has long been identified in many cratons [20,22] and strongly relate to the geochemistry of granitoids ([37], figure 1), which are thus a prime Archaean archive. The oldest granitoids are intermediate to felsic intrusions of the sodic tonalite–trondhjemite–granodiorite series (TTG, predominantly defined by high Na/K) (table 1) [38]. Globally, intrusions with this distinct chemical character progressively diminish towards the Late Archaean and Palaeoproterozoic and are succeeded by intrusions with increasingly potassic character [37,39,40]. Processes, volume or chemical definitions vary between cratons and authors though, and leave much room for interpretation.

**Table 1.** Definition of terms used in this paper.

term	definition in this contribution
TTG	tonalite–trondhjemite–granodiorite; a plutonic rock series that is characterized by high Na_2_O/K_2_O;
	towards the Mid- to Late Archaean elevated Sr/Y and heavy rare-earth element depletion through retention in residual garnet and/or amphibole during partial melting become increasingly common
	proposed to be derived from melting of (hydrous) mafic successions
	trondhjemites versus tonalites may be a function of pressure of melting
transitional TTG	similar to TTG, but with a spectrum of K content intermediate between TTG and ACP granites
	within Archaean Domes typically offset from TTG melts by 100–300 Ma, in the North Kaapvaal craton of South Africa often dispersed within TTG gneisses
sanukitoids	potassic diorites with elevated Mg, Cr and Ni;
	Proposed to have formed by interactions of TTG melt with peridotite. The sanukitoids s.s. are the root of a series of more differentiated rocks (sanukitoid suite)
ACP granites or high-K granites	Archaean crustal progeny granites, formed by remelting of earlier, silicic crust. Plutonic rocks with a markedly elevated K content compared to TTG. Plausible petrogenetic models to create elevated K contents granitic melts towards the Mid- to Late Archaean argue for a re-melting of earlier TTG succession. For this, petrologic phase equilibria require either excess heat in TTG-bearing crust, or elevated pressure–temperature through subsidence
stagnant lid	the entire lithosphere of the planet that covers the mantle as a lid without active tectonics. Sporadic subduction is possible; the lid is characterized by felsic intrusions into a stationary lithosphere
(down- or lithospheric) drips	drip of the lowermost lithosphere into the underlying mantle, initiated by converging convection cells. These drips have a limited lifespan of several hundreds of millions of years and occur randomly between rather small convection cells
downwellings	similar to drips, but with a substantially longer lifespan and stationary at the interface between thick and thin plates, i.e. a proto-craton and a proto-plate
lithosphere	the outermost shell of the planet, including the crust and its underlying, non-convecting mantle
lithospheric mantle	the rigid, non-convecting part of the lithosphere, underpinning the crust. In post-Archaean time, this is often termed sub-continental lithospheric mantle
proto-craton	former part of a stagnant lid regime that thickens due to melt injection and intra-crustal flow. This part is today preserved as cratons and can date back to the early Archaean, even with Hadean components
proto-oceanic crust	former part of the stagnant lid that thins out though thermal erosion, e.g. via plumes, or through intra-crustal flow. Proto-oceanic crust have been destroyed through subduction, with modern oceanic crust as analogues

In this contribution, we propose a geodynamic model that accounts for the shift in granitoid chemistry between 3.0 and 2.5 Ga from TTG towards granites with higher K contents (often referred to as high-K granites, but termed here *Archaean crustal progeny granites*, or for convenience ACP granites, cf. table 1). We use numeric modelling to investigate lithospheric conditions in the Archaean and to map out intra-lithospheric flow. Changes in crustal thickness and the response of the lithospheric mantle between crust and convecting mantle lead to the sequential development of key rock types of the Archaean period. Based on the sum of observations, we propose a geodynamic model, which illustrates a range of chemical features in the transition from the Archaean to the Proterozoic era. Importantly, this model allows individual crustal segments to follow parallel evolutions, at different times, thus reproducing the important observation that the global Late-Archaean transition proceeds from the accumulation of changes on a regional scale. This regime bridges the evolution from a non-plate tectonic mode (or stagnant lid) towards mobile plate tectonics.

## 2. Background to Archaean tonalite–trondhjemite–granodiorite intrusions

Tonalites, trondhjemites and granodiorites comprise a range of plutonic rocks of predominantly Archaean age [41]. A number of recent review articles covers their definition, compositional range and genesis [37–40,42]. Because the terminology of these rocks and terms associated with their genesis is often confusing, table 1 contains a range of definitions used in this contribution. In brief, TTG are phaneritic rocks that contain quartz, have plagioclase as the main feldspar, and in the case of trondhjemites (or plagiogranites), the plagioclase is Na-rich, and overall they have a relatively low K for a given Na content. This is suggestive of formation by melting of a (meta)mafic source. The striking feature of TTG is their depletion in heavy rare earth elements (HREE); a geochemical signature assigned to residual mineralogy during partial melting [43,44]. This depletion requires a pressure regime of melting between approximately 1 GPa [45,46] in the crust to greater than 3 GPa at mantle depths [47,48].

In detail, Drummond & Defant [49] observed elevated Sr/Y and fractionated light from HREE (high La/Yb) in many TTG. Both proxies are fractionated by, and are thus indicative of, residual garnet and/or amphibole, which is often used as an indicator for melting at a pressure of *ca* greater than 1 GPa (greater than 25 km of crust). However, garnet stability and mode is very strongly dependent on crustal rock composition and can occur at pressures as low as 0.7 GPa (equating to *ca* 20 km of crust) for low-Mg meta-basaltic successions and a high geothermal gradient [46,50,51], such that more enriched mafic rocks are able to yield high Sr/Y and La/Yb at pressures lower than depleted mafic rocks [42,52]. Indeed, the melting residue of many (enriched) granulite-facies metamafic rocks in deep crustal conditions contains significant amounts of garnet, such that the matching melt had high La/Yb and Sr/Y ratios [53]). Likewise, low Nb/Ta and Nb depletion in Archaean TTG have been interpreted as high pressure (*ca* 3 GPa) melting of depleted mafic rocks, with rutile in their residue [47]. However, a source with a lower Nb/Ta, such as a low-Mg amphibolite, would yield low Nb/Ta melts without the need of large amounts of rutile—and thus without the need for high pressure [54,55].

Similarities to modern-day partial melts of the subducted oceanic crust e.g. in Southern Chile [56] have led to the perception of TTG being Archaean analogues of adakites representing melting of oceanic crust in hot subduction zones [41,57]. This was consistent with the perceived requirement for high pressure melting, as outlined above. However, this largely relies on the assumption of a depleted mafic source. If the source of TTG was similar to common Archaean basalts, that are less depleted (e.g. [36,58]), the depth requirement is significantly relaxed [59] and the case for subduction is less compelling. Based on this line of evidence, models such as delamination and partial melting of oceanic plateaux [60] or sagduction [53] become more tenable. An additional, important clue on the source material of TTG melts is preserved in δ^18^O_SMOW_ isotopes in zircons, some of which have values of up to 7‰, well above mantle values [61]. Near-surface weathering of rocks best explains this feature [62], in line with partial melting modelling of hydrated meta-volcanic rock [36,51]. Furthermore, many TTG contain abundant mica and are thus crystallized from melts with a considerable water content. Without any inference for a geodynamic regime, this demonstrates that burial of mafic rocks that have undergone surface alteration is required to bring the source rocks of TTG into regions of partial melting in the presence of garnet (greater than *ca* 1 GPa or 25 km for enriched rocks, and even higher pressures for less enriched compositions) [50].

Unrelated but important is the recognition by Martin & Moyen [44] of a seemingly temporal decrease in combined Na + Ca and Sr in TTG from Early to Late Archaean time. Subsequently, it was noted that high-Sr TTG appears progressively with time [36,40], while low-Sr TTG (i.e. low pressure melting in the presence of plagioclase) remain constant throughout the Archaean. The inference is that the spectrum of TTG compositions (and therefore, presumably, the variety of depth of melting and geodynamic environments) progressively widens during the Archaean. The increasing importance of high-Sr TTG demands a more prominent role of melting in the presence of larger amounts of garnet (i.e. deeper, high-pressure melting for a given source composition) towards the Late Archaean. Possible reasons are a growing crustal thickness with time, or geodynamic processes that allow melting at increasing depth, e.g. in drips or through some sort of subduction. This important observation indicates a temporal evolution in the modes of melt production within the crust and that TTG are not a simple, single type of melt but rather a pool of rock types with a spectrum of compositions. Any model that includes the chemical composition of TTG through time thus needs to account for changing conditions of melting of crustal successions, whether they are buried or subducted. Hence, while this highlights a temporal evolution of melt chemistry in general, complications may further arise through spatial variations between cratons, and even within cratons; a fact that is often not accounted for, simply because of the difficulties in reconstructing spatial relations of TTG in remnants of highly modified Archaean crust.

The geodynamic framework for all of these changes remains equally confusing and unresolved. One possible scenario for achieving burial of near-surface igneous rock is through a continuous volcanic overload [63]. A compelling alternative, based on geodynamic modelling, and which differs from chemistry-based subduction models, is melting of meta-basalt in drips that consist of negatively buoyant crustal blobs that sink into the mantle [53,64]. The rigid, non-convecting lithospheric mantle between the crust and convecting asthenospheric mantle (in post-Archaean time often termed sub-continental or sub-oceanic lithospheric mantle) plays a fundamental role in these models. Mantle convection can potentially induce horizontal stress sufficient to initiate crustal extension or compression [65]. Parameters deemed most favourable in these models include prior, plume-triggered erosion of the lithospheric mantle [66,67] and crustal thickening by intra-crustal flow in which lithospheric mantle material is dragged into a convecting maelstrom [53]. Given the abundant komatiitic successions in Archaean greenstone belts and the association of komatiites with plumes or hot spots [68,69], this assumption seems plausible.

## 3. Archaean crustal progeny granites and sanukitoids

In each craton, the geodynamic stages before reaching craton stabilization, as indicated by dike-swarm emplacement and large intracratonic basin deposition, are marked by the emplacement of granitoids that are chemically different to TTG [37,70]. All of these Archaean granites have elevated K, yet without clear definition. These granitoids feature two fundamentally different suites of rocks with one group requiring predominantly crustal reworking, whereas the other is dominated by a more mafic, mantle-derived input. The former is termed here ACP granites, the latter sanukitoids. Below, we will give a brief overview of how these differ from each other and from TTG.

ACP granites feature reworking of felsic lithologies, which is recorded in granitoid compositions with increasing concentrations in large ion lithophile elements (LILE, such as K, Rb, Ba and Th) [71]. From a major element perspective, this equates to an increase in K_2_O (or K_2_O/Na_2_O), and classifies these rocks as granites, as opposed to TTG. Striking is that these granites are similar to TTG in their REE patterns, although they tend to develop a small, negative Eu anomaly that is mostly absent from TTG. Most of the ACP granitoids are mildly peraluminous (1 < A/CNK < 1.05), similar to TTG, but unlike modern arc granitoids that are typically metaluminous (0.9 < A/CNK < 1.05). It is notable that occasionally some ACP granites qualify as S-type granites with muscovite and/or cordierite and/or garnet, and A/CNK of up to 1.3. Assuming these originated, similar to modern S-types [72] by melting of sediments, such Archaean S-types represent the extreme endmember scenario of a crustal reworking process. Unravelling the melting conditions of the source of ACP granites is not straightforward. Archaean granitoids define a continuous range of composition from TTG over transitional TTG to ACP granites (elsewhere referred to as high-K or biotite-granites [37]). The continuous nature of the range of compositions suggests either that all possible sources, from more mafic to more felsic, were tapped to form these granitoids, or that the sources were mixtures, in all proportions, of mafic and felsic (or older TTG) components. The latter is our preferred scenario based on trace element systematics: if their source included (or was even dominated by) a TTG component, reworked ACP granites inherited the high-pressure signature of their precursor TTG. The mild Eu (and Sr) anomalies are consistent with melting in the presence of plagioclase, and the fact that REE patterns are not much more fractionated than those of TTG suggests that garnet was not an abundant or dominating residual mineral.

Granitoids with a mantle component, or sanukitoids, cover a very diverse group with no clear chemical definition [37] and may have formed by a range of petrogenetic processes that include melting of a variety of sources, igneous differentiation, assimilation of country rocks, and/or mixing with crustal melts at emplacement depths. However, all rocks from this group share some common features, that is a potassic, yet mafic to intermediate (51–55% SiO_2_) parental magma. These sanukitoid diorites are invariably high-MgO and high-Mg# melts and enriched in incompatible elements, most notably Ba and Sr, and sometimes high-field strength elements (HFSE) [73]. A most popular explanation is that they originated from partial melting of an enriched mantle source [74–76]. In this scenario, the enrichment in both LILE and HFSE suggests mobility of both groups in silicate melts rather than fluids, in contrast to modern arc granitoids [36]. Although this observation has been used to support an arc model (e.g. [38]) with mantle–melt interaction occurring in the mantle wedge above a slab, we emphasize here that the only requirement is a stratified geometry that allows felsic melt to interact with overlying peridotites [36,77].

A clear distinction can be drawn between these two types of Archaean granitoids and modern active continental margin intrusions. Although the latter are often classified as potassic granitoids, they are typically amphibole-bearing and metaluminous, features that are non-existent in the Archaean. Modern arc intrusions further lack HFSE enrichment of sanukitoids, and are moderately LILE-enriched only (i.e. dwarfed by the Ba–Sr enrichment in the Archaean). It is further intriguing that transitional TTG often predates or ceases contemporaneously with the first appearance of ACP granites. Sanukitoids sometimes occur contemporaneously with this change towards ACP granites but are volumetrically small in Archaean terranes (figure 1). This consistent, secular evolution of intrusion chemistry requires a geodynamic explanation that is valid for all cratons.

**Figure 2.**
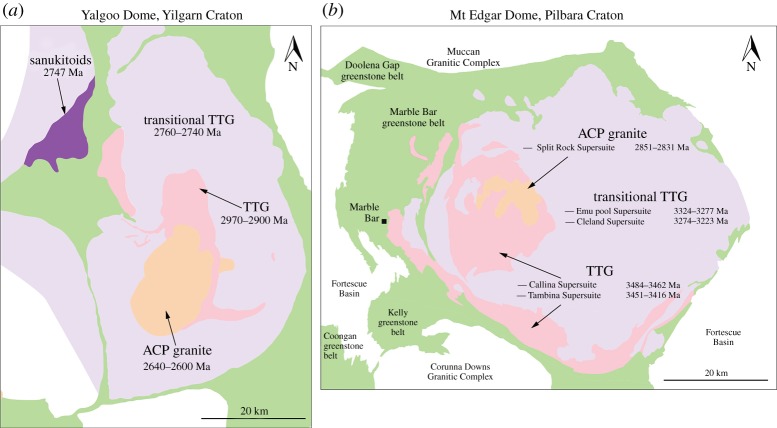
Geological map of (*a*) Yalgoo Dome and the (*b*) Mt Edgar Dome of the Australian Yilgarn and Pilbara Cratons, respectively in Australia [81]. Both domes contain a centre of old TTG melts, surrounded by larger volumes of transitional TTG with elevated K contents (figure 3 for details about the elevated K contents). The youngest unit in both domes is a high-K granite, located in the centre of the dome. The structure is proposed here to reflect a birds-eye view onto a fossil drip. In the case of the Yalgoo Dome, late-stage, volumetrically small sanukitoid melts are located on the outer rim of the dome, which is proposed to reflect diversion of the drip from a vertical towards a tilted geometry that allows melts to pass through an overlying mantle (cf. figure 5). (Online version in colour.)

**Figure 3.**
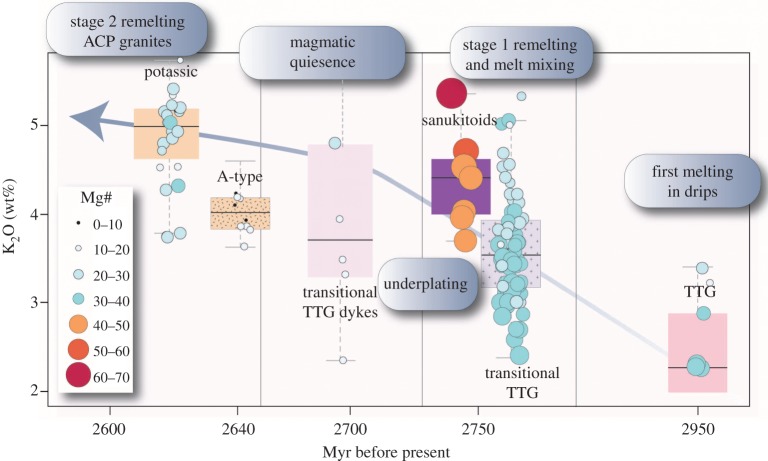
The shift in chemistry from proto-subduction TTG over transitional TTG towards K-rich granites [81] preserved within the Yalgoo dome in the Australian Pilbara Craton. This dome reflects crustal evolution from TTG melts towards ACP-granites within a timespan of *ca* 200–300 Ma with intermediate melts of transitional TTG. The latter are followed by low volume sanukitoids, characterized by elevated, mantle-derived Mg-Cr. The Mg# is the molar Mg/(Mg + Fe) of a rock. (Online version in colour.)

**Figure 4.**
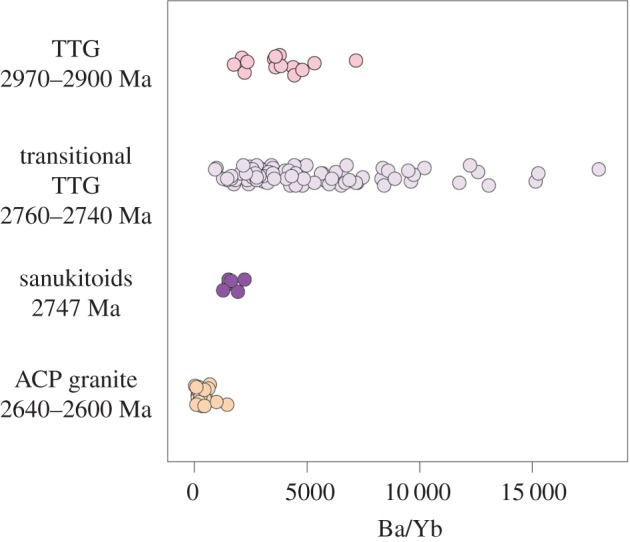
Ba/Yb in igneous rocks, divided according to intrusion episodes, of the Yalgoo Dome illustrating the increase in LILE in transitional (tr) TTG. The increase is only visible for this rock suite, and is proposed to reflect the role of partial melting of plagioclase and/or mica. (Online version in colour.)

In a nutshell, this global or craton-wide evolution, albeit not occurring contemporaneously worldwide, is recorded in dome structures in the Yilgarn craton. The Yilgarn craton, and its Murchison terrane in particular, preserves a range of plutonic rocks from greater than 3 Ga to less than 2.5 Ga [78,79], which based on the statistical geochemistry of available global data [27], corresponds with the proposed change in the average chemical composition of the crust. A key advantage of the Archaean Murchison terrane is that crustal chemistry is preserved on a small, tens of kilometre scale in what appears to be defined dome structures [80]. For example, the temporal change in intrusion composition is well demonstrated by the Yalgoo or Mt Edgar Domes in the Yilgarn and Pilbara Cratons, respectively (figure 2). Within the dome, TTG evolves to higher K granitic rocks over time-scales of *ca* 300 Ma, from an old TTG core, towards volumetrically larger TTG with higher K content (termed transitional TTG here), to final, ACP granites (figure 3). These transitional phases display slightly elevated K_2_O/Na_2_O and sometimes higher Ba content (figure 4), but otherwise typical TTG trace element patterns. This transition to higher K contents in granitoids occurs in conjunction with the appearance of sanukitoids.

## 4. Geodynamic framework for tonalite–trondhjemite–granodiorite melts

The geodynamic framework for the melting of Archaean crust to form TTG requires subsidence of crustal assemblages probably into the garnet stability field prior to partial melting to generate their characteristic REE pattern. In an Archaean Earth in which subduction, as we see it today, is either absent or at best sporadic [10,15,82–84], a mechanism needs to be identified that drives subsidence. Computational simulations using presumed geodynamic parameters of the Archaean mantle and crust found that the convecting mantle under a crustal lid creates lithospheric drips into the mantle [53,85]. This model is an efficient way to bring crustal successions to mantle depths and allows for partial melting of this material in an elevated P-T environment. Here, we link this geodynamic model with the geochemistry of preserved Archaean granitoids, enabling the subsidence of Archaean basaltic rocks and their melting to form TTG.

**Figure 5.**
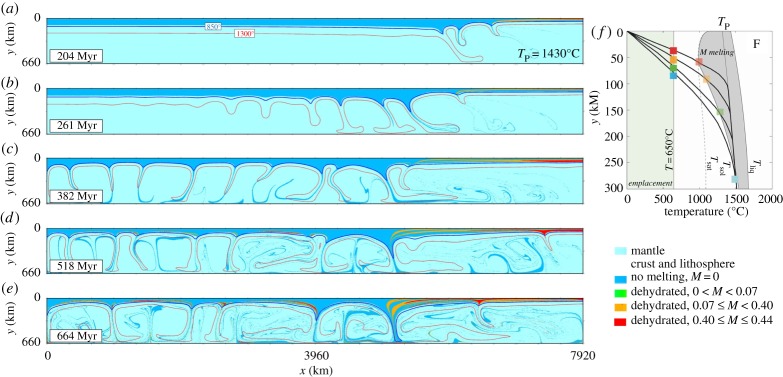
Numerical model of mantle convection and melting. Model shows two coexisting regimes: proto-plate on the right and stagnant lid on left. Material is shown (colour-coded) and tracked as it moves from an undifferentiated asthenospheric mantle (light blue), to the lithosphere and crust. Progressive melt production *M* is calculated from adiabatic mantle temperature and pressure, and shown as no-melt bearing lithosphere and crust, *M* = 0 (blue), and melt-bearing lithosphere and crust with 0 < *M* < 0.44 (turquoise, green and yellow). For clarity colour coding is applied when reaching *T* < 650°C, when the melt-bearing rocks are considered emplaced in the crust. The model has mantle potential temperature *T*_P_ = 1430°C, adiabatic gradient of 3°C/km, and initial mantle hydration of *X*_H_2_O_ = 0.2 wt%. The imposed initial lithosphere thinning on the 330 km on the right of the model is Δ*h* = 20 km measured along the 650°C isotherm. (Online version in colour.)

Computational models have proven to be useful to simulate the regimes of the Earth's past and the evolution of Archaean crust [86]. Our model implements a geodynamics framework with open-source code (Underworld [87]) and is derived from laboratory-constrained rheological laws [88] and melting parametrization [89,90], which are commonly used in published numerical models of the Archaean mantle (e.g. [91]). The model is initiated with a thick thermal boundary layer (analogous to the lithospheric mantle), which develops steady-state convection of the mantle under a stagnant lid (illustrated in figure 4). In our approach, we use a 660 km deep section, reproducing the upper mantle, with a width of 7920 km. The initial thickness of the lid is chosen to vary across a horizontal distance of approximately 330 km (on the right in figure 4), simulating the effect of a plume impinging on the lithosphere [64]. Mantle potential temperature of 1430°C and initial mantle hydration of 0.2 wt% are chosen, compatible with what is inferred for the Archaean [14,92,93], and an adiabatic temperature is calculated for the melting fraction *M* (figure 5). Modelling the melting and emplacement of melt-bearing rocks in the crust (defined for *T*  <  650°C) simulates the differentiation of mantle rocks into basaltic crust.

**Figure 6.**
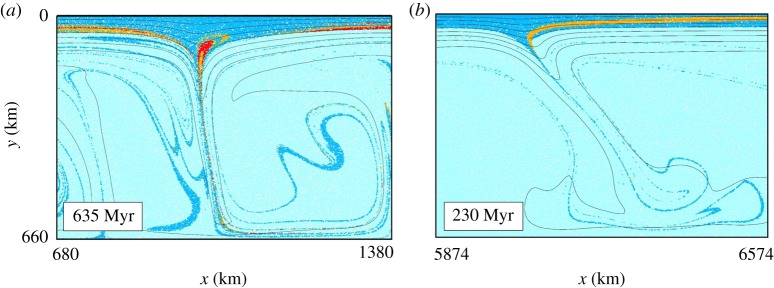
Detail of numerical model with isotherms and composition. Legend similar to figure 1. (*a*) Vertical drips beneath a stagnant lid form, where deeply emplaced melt is recycled in the mantle and (*b*) lithosphere migration towards the left of the model forces drips into asymmetric downwellings, where rocks containing weathered basaltic successions are taken from surface to depth in a subduction-like environment, yet with no subduction. (Online version in colour.)

In essence, the model creates a crustal lid of variable thickness, illustrates subsidence and material movement within the Archaean crust, and pictures loci of generation and spatial distribution of magma within the crust. This model, similar to others before it, illustrates a range of features, which are comparable to Archaean tectonics. Part of the model illustrates crustal lithosphere that evolves into a thicker, stagnant lid domain (figure 5, towards the left), where vertical drips form beneath a rather immobile lithosphere and a more mobile, thinner lid. Within the stagnant, thicker portion of the lid, volumetrically significant melt is produced within drips and this section can be traced for approximately 300 Ma, when it is dragged back into the drip by mantle convection periodic overturns (figure 6*a*). In the other, thinner mobile domain (right in figure 6*b*), the thinning of the lithosphere allows shallow melting within the lithosphere giving rise to internal differentiation. Lateral flow of this lithosphere progressively transports these differentiated rocks into areas of downwellings. The migration of these sections into one preferential side of the downwelling results in asymmetric, subduction-like tilted downwellings, which may persist for approximately 500 Ma (figure 6*b*).

Key to the model is that under stagnant lid starting conditions, regions above up- and downwelling zones in the convecting mantle will develop pronounced variation in lithospheric thickness [94] (and observed in figure 5*a* towards the right of the box). Elsewhere, plume-related volcanism in the Archaean will enhance crustal thickness through voluminous deep intrusions [95], shallower sills, and extruding lava but also possible lower lithospheric thinning through thermal erosion [96–98]. A second important aspect of the model is partial melting of the lithospheric mantle underpinning the crust. Melting of the Archaean mantle is evident through the rock associations in Archaean greenstone belts and cratonic cores of modern continents [99,100]. It has further been argued that mantle melting will have a profound effect on the rigidity of this crust [101]. While extruding melts add to the chemical differentiation of the crust, they also leave depleted, residual counterparts behind that form rigid, high-viscosity regions. Such residues, possibly harzburgitic in nature, are Fe-depleted, or artificially enriched in Mg, and so positively buoyant compared to ambient lherzolite. Melting of the lithospheric mantle that has undergone depletion and re-enrichment throughout its history may not only account for the diversity of Archaean basaltic rocks [102], but also the stability of the overlying crust through a progressively melt-depleted lithospheric keel. In our model, this melt-induced crustal differentiation (or production [101]) and associated flow in response to the change in stiffness can be achieved on length scales of 300 km and may persist for up to approximately 500–600 Ma (though using higher temperatures these time scales become shorter). It seems plausible to relate the thicker lid regions (the central domain in figure 4) with cores of cratons that are preserved today, whereas the lower, thinner crustal areas are likely not preserved today and have been destroyed through later subduction. Isostasy demands that the thicker crustal regions form lower crustal successions with the underlying lithospheric mantle that would most certainly interact with the underlying convecting mantle. Smaller crustal drips are generated seemingly randomly underneath these proto-cratons, and larger, downdwellings are created at the interface between thicker crustal proto-cratons and thinner crust. Either could be of short duration or long-lasting, over time scales of greater than 400 Ma (figure 5).

Support for such drips is given by the model of Johnson *et al*. [53], who speculated that lithospheric roots could potentially detach and become incorporated into the upper mantle's convecting regime. Dense, residual successions could detach and sink as cold-drips into the lower mantle [103]. Based on isotope evidence in komatiites that are sourced from a material that originates in the deep mantle sections, these eventually return to the surface during later mantle plume activity [66]. These authors referred to this as an early refractory reservoir residing in and cycling through the early mantle in a plume-driven convection cycle. This inevitably requires a melt-depletion of these successions, which is related here to the formation of TTG and explained further below.

In summary, and in relation to the abundant Archaean TTG successions, it seems plausible to relate the crustal drips to partial melting of entrained crust, and we propose a scenario here in which felsic TTG melts are formed within these drips. Several observations support this hypothesis. Firstly, lateral transport of material within the crust aids the transfer of material with variable stiffness from close to the surface towards the base of the crust (figure 6*b*). Similar to earlier models [53,64], this intra-crustal flow provides a feasible mechanism for deep crustal burial of former near-surface material, and with this hydrated meta-basalt. For the genesis of TTG, this is required following oxygen isotope systematics in zircons derived from TTG, which indicate that weathered material is a key prerequisite for their genesis [62]. In addition, hydrated basalt was highlighted previously as a potential precursor material for TTG [36,51]. Secondly, the model shows that the convecting Archaean upper mantle gives rise to temporary, relatively focused areas of downward flow. Some drips appear to be stationary in/over a convecting mantle and may last greater than 300–400 Ma, which is plausible in a regime without moving plates, and coherent with the intra-dome temporal evolution from TTG to ACP granites in the Yalgoo Dome (figure 3). The distribution of such drips is defined by the palaeo-topography, which effects the negative topography of crust through isostasy and that of the pattern of convection. It is proposed here that the products of melting from these drips that rise and intrude into the overlying crust are preserved in today's cratons as domes. In other words, such domes represent a crustal ‘bird's-eye’ view onto these drips. Despite being distributed in a seemingly random pattern, similar dome structures are found in the Murchison domain of the Yilgarn Craton. In such an area, a crustal yoyo is proposed: areas of drips melt, inject melt into overlying crustal successions until these themselves are dragged into the drips and are partially melted. This can then explain the elevation of K through crustal cannibalism. In other proteo-cratons, no domes of this sort are observed, e.g. in the Kaapvaal craton. Following our modelling results illustrated in figure 5, the measured temporal variations in the duration of TTG magmatism highlight that some drips (and associated melts) are stationary for protracted periods, whereas others are transitory.

## 5. Tonalite–trondhjemite–granodiorite melt evolution within drips and domes

A key aspect of the temporal evolution of felsic melts that are produced in the Archaean crust is that of the maturation from TTG to transitional TTG. This change is most profoundly monitored in the gradual elevation of absolute K content (figure 3) or in increasing K/Na, notwithstanding the possibility of changes in other geochemical proxies related to this. If it is accepted that the formation of TTG melts is the result of partial melting of meta-basaltic successions within drips [51] and that the temporal evolution of granitoid chemistry within domes, such as the Yalgoo and Mt Edgar Domes (figure 2), reflects the evolution of these drips, then the chemo-temporal evolution of each individual dome reflects at least two periods of re-melting of crustal successions. In a nutshell (or a dome), the change from TTG melts towards transitional TTG within drips is best explained by a model in which, in a second stage of melting, original TTG melts (derived from different meta-basaltic successions) are combined with melts from re-melted, existing TTG. In the Brazilian São Fransico Craton, the transition towards elevated K in TTG melts was explained by this process [104]. The TTG in the African Congo Craton follow a similar path [105]. The mix of both melts creates a hybrid melt blend producing compositions between a TTG melt and a high-K granite endmember, which can account for the large spectrum of K contents (figure 3). These hybrid melts form transitional TTG that sometimes do not mix perfectly and are preserved as mingled plutonic bodies. This can readily explain the occurrence of different petrologic successions of seemingly similar age in close proximity (or within one plutonic complex). This hybrid model follows a logic step in drips, where TTG are produced but when dragged back down into the drip, re-melt together with other basaltic material. In the Yalgoo dome, this hybrid mix is accompanied by elevated Ba contents in the transitional TTG. High Ba in granitic rocks most certainly reflects the presence of mica or plagioclase in their source region. Transitional TTG within the Mount Edgar Dome, however, do not share this feature, which further highlights the chemical diversity among TTG as a function of localized settings, and also that trace element abundances must be treated with caution as indicators for geodynamic processes. It is not clear from field evidence, geochemistry or modelling, however, why older TTG melts are followed by an episode magmatic quiescence (figure 3) before the development of transitional TTG. The pattern, however, seems to be coherent.

**Figure 7.**
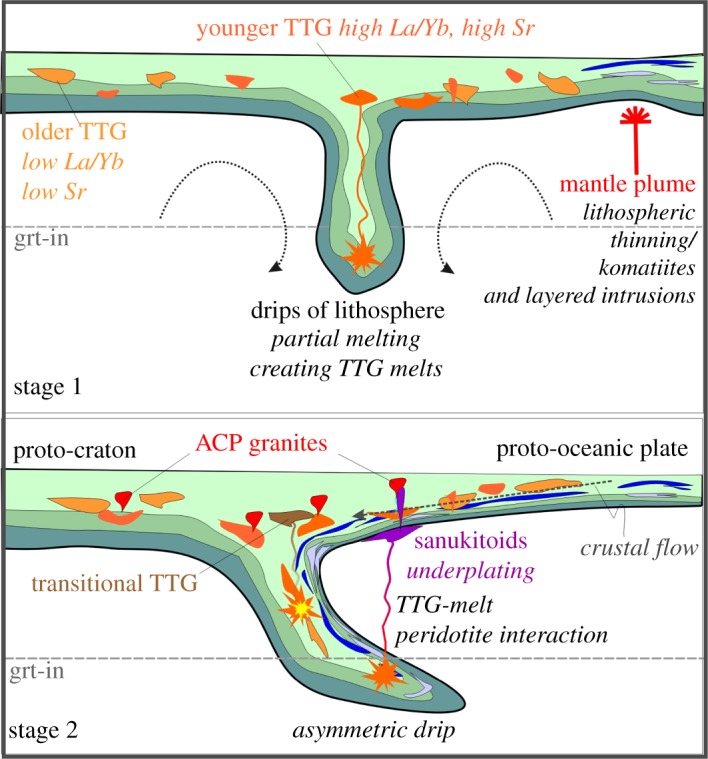
Schematic sketch of (*a*) Lower crustal drips with partial melting of submerged crust, giving rise to TTG. Drips occur randomly and are driven by convection of the underlying mantle; mantle plumes erode the lithosphere and inject layered intrusions and komatiitic melts. Older TTG form at shallower levels in the presence of plag, younger TTG in the presence of crustal grt. (*b*) Transitional TTG will form within drips as blends of melts derived from existing TTG and meta-basalt. Asymmetric drip or downwelling are stationary drips that develop over prolonged periods of time at the interface between thicker and thinner crustal planes. Through intra-crustal flow, asymmetric drips can develop through differences in crustal stiffness. The tilt will force rising TTG melt to pass through the overlying mantle, forming sanukitoids and predominantly accumulate at the base of the crust. Excess heat introduced through this underplating gives rise to partial melting of existing TTG to form ACP granites. Thicker plate regions represent proto-cratons, thinner plate regions proto-oceanic crust. At their interface, subduction eventually starts. Within stages, no temporal evolution is given. (Online version in colour.)

The final stage of melting within drips appears to be dominated by low-volume ACP granites, sometimes accompanied by (high-Mg) sanukitoids [106]. The ACP granites lack residual garnet signatures, and it appears most plausible to ascribe these to partial melting of existing TTG and potentially even reworked crust in the form of mica-bearing sedimentary rocks, adding a potential S-type component to these rocks. While the lack of a garnet signature signals a shallower melting, these hypothesized parental rock associations also have a lower or similar melting point compared to meta-basaltic rocks or amphibolite (i.e. 700–800°C versus 900°C [46]). What is thus required is a thermal pulse into the crust in areas where drips have developed previously. The key to this question maybe the longevity of drips in the same location, or, alternatively, the rare occurrence of sanukitoid melts. Sanukitoids, or high-Mg diorites [106], can potentially be explained by the geodynamic of drips with a non-uniform chemical composition. Crustal successions that are dragged into the mantle through convection will develop an asymmetry caused by variations in stiffness (figure 7). Naturally, parental melts that are produced in an angular downwelling are identical to other TTG melts. The subsequent vertical ascent of the melts towards the surface, however, requires the melt to pass through mantle-peridotite. In other words, TTG melts that otherwise would rise into the overlying crust are now forced to migrate to the surface through the overlying mantle. This process is, from a petrologic point of view, not dissimilar to a slab-melting scenario in a subduction zone, yet is placed in a lid-tectonic geodynamic framework. It has been shown experimentally that TTG melt-peridotite interaction produces melts of sanukitoid composition [75]. Melt-rock reactions increase Mg, Cr and Ni to form high-Mg dolerites. In Archaean terranes, sanukitoids are volumetrically small but occur constantly among global cratons, shortly before or contemporaneous with ACP granites.

It has long been known that melts which pass through the mantle do not readily invade the overlying crust but stall at its base [107], with some melts intruding into the overlying crust [108]. In such a scenario, where sanukitoids originate within asymmetric drips, these melts constitute a critical medium for heat transfer into the overlying, lower Archaean crust. By means of underplating, these hot melts will form part of the lowermost crust, not dissimilar to modern convergent continental margins. In modern subduction environments, where melts pass from the mantle towards a thick arc crust, it is suggested, by means of mass balance, that the majority of I-type granites or batholiths are likely (but not inevitably) generated by the re-melting of lower arc crust through this thermal spike, rather than by direct injection of mantle-derived melts into the crust and subsequent igneous differentiation [109]. Projecting this model to the Archaean, a different yet equally plausible mechanism is plutonic melt generation through re-melting of TTG-bearing crust by heating through sanukitoid underplating. In this scenario, the majority of sanukitoid melts do not enter the upper to middle crust, but remain at the base of the crust and act as a thermal patch at the base of the crust.

## 6. Towards a unified model

A major challenge in unravelling the Archaean Earth is to integrate geodynamic and geochemical concepts of early crustal formation into a unified model. Here it is proposed that the evolution of crust is initiated by the response of a mobile *and hydrated* lithosphere to an underlying, convecting mantle. A key concept in this model is that of long-lived lithospheric drips, which lead to abundant TTG melting in a squishy, mobile or deformable crustal lid [95]. Such drips have been created by computational models elsewhere e.g. [53,85] and, if correct, add a fundamental aspect to the geodynamic melting regimes in the Archaean that does not require modern-style subduction (even though it does not exclude it).

**Figure 8.**
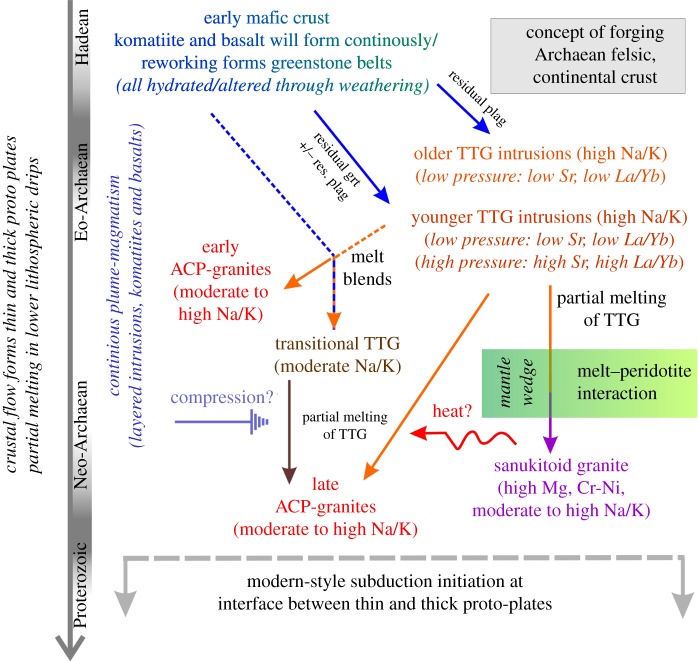
Concept of melt generation in a broad temporal context on the Archaean Earth. The sequence of events here is suggested based on the chemical evolution of granitoid melt compositions through time and the computational modelling of the evolution of Archaean crust. Grt, garnet; plag, plagioclase. Note that time is not linear. (Online version in colour.)

In our preferred scenario, illustrated in figure 7 and sketched as a concept in figure 8, initial partial melting of the upper mantle is triggered through plume activity and crustal thinning, which results in near-surface intrusions or extrusive lavas of komatiitic to basaltic composition. This process will add to crustal thickness, differentiation and variable stiffness. Intra-crustal or lithospheric flow can allow for the burial of material that formerly resided close to the surface, including these basaltic layers. This material would need to be hydrated, in order to satisfy isotope observations (e.g. oxygen isotopes), allow for easier partial melting (e.g. dehydration melting of amphibolites in the absence of free water), and is backed by amphibolitic xenoliths in many TTG outcrops. Crucially, entrainment of this (partially hydrated) material into lower lithospheric drips then allows for further subsidence of this basaltic material and partial melts thereof are parental to TTG. With time, subsidence of existing TTG within long-lived drips, or simply by crustal thickening, will result in hybrid melts between TTG compositions derived from basaltic succession and melts from existing, older TTG, forming transitional TTG. This evolution may be related to long-lasting drips and form dome structures, as is the case in the Australian Yilgarn craton, or more spatially dispersed intrusions, as can be observed in the Barberton area in South Africa. These intrusions even promote a thermal regime in the Archaean crust that would be in itself beneficial to maintaining the process [95].

Subsequently, drips may develop an asymmetric shape at the interface between thickened lithosphere (proto-cratons) and thinned lithosphere (proto-oceanic plates), which are consequent to lithospheric flow that was driven by convection. This bi-modal plate regime is a key parameter in developing plate tectonics. The form of the lithospheric asymmetries are reminiscent of subduction zones but are driven by relatively rapid mantle convection and not by the potential energy associated with the density instability of oceanic lithosphere in a plate tectonic regime. TTG melts derived from asymmetric drips rise through and react with the overlying mantle. The resultant rocks, termed sanukitoid TTG, are high in Mg-Cr. We speculate that these melts stall at the base of the overlying crust by a density contrast, are underplated, similar to modern style, subduction zones melts, and thereby inject a thermal pulse into the lower crust that allows re-melting of existing TTG-bearing crust. Naturally, by their density contrast, very few if any sanukitoid melts are injected into the crust, which can explain their scarcity in Archaean terranes. These melts seem to globally pre-date ACP granites [26], so that a genetic relation seems plausible.

An inevitable conclusion of the above model is that while melting within a drip-dome system is following a temporal pattern towards higher K contents, these evolutions are confined to each drip (today preserved in a tectonic dome). It appears that each dome follows the same schematic evolution with increasing K contents from a first stage of pure TTG melts towards a second stage with transitional TTG towards ACP granites, ±sanukitoids over a time interval of *ca* 100–200 Ma (figure 3). However, because of the random spatial and temporal distribution of drips, they do not occur contemporaneously so that a craton-wide evolution cannot be reflected in a chemical survey of pooled datasets (figure 1). The same applies where drips develop more randomly and do not form domes. This is important when large geochemical datasets are used to constrain crustal evolution.

All of these processes do not require active plate tectonics, so it remains enigmatic why drip tectonics converted into active subduction zones. One possible solution is the development of substantial downwellings at the interface between thicker and thinner areas of the lithosphere. The thermal pulse of sanukitoid melts in these areas would add to the weakening of the overlying crust. Changing lithospheric thickness and crustal movement in response to mantle convection eventually leads to a break in the lid and the lateral transfer and thrusting of regions of thinned lithosphere under the proto-cratons.

The feasibility of this model can be tested by further detailed field observations, more sophisticated modelling attempts, based on, and in conjunction with, field data, and the analyses of various geochemical proxies on the same rocks. Since it is often not possible to integrate all of these features into a single study or hard to comprehend the complexity of multiple disciplines in a single study, what is suggested here is that our model serves as a baseline against which approaches from various disciplines can be tested. This should lead to refined models that should further approach reality.

## Data accessibility

This article has no additional data.
